# RNA-Seq analysis of gene expression for floral development in crested wheatgrass (*Agropyron cristatum* L.)

**DOI:** 10.1371/journal.pone.0177417

**Published:** 2017-05-22

**Authors:** Fangqin Zeng, Bill Biligetu, Bruce Coulman, Michael P. Schellenberg, Yong-Bi Fu

**Affiliations:** 1 Department of Plant Sciences, University of Saskatchewan, Saskatoon, Saskatchewan, Canada; 2 Swift Current Research and Development Center, Agriculture and Agri-Food Canada, Swift Current, Saskatchewan, Canada; 3 Plant Gene Resources of Canada, Saskatoon Research and Development Centre, Agriculture and Agri-Food Canada, Saskatoon, Saskatchewan, Canada; Saint Mary's University, CANADA

## Abstract

Crested wheatgrass [*Agropyron cristatum* L. (Gaertn.)] is widely used for early spring grazing in western Canada and the development of late maturing cultivars which maintain forage quality for a longer period is desired. However, it is difficult to manipulate the timing of floral transition, as little is known about molecular mechanism of plant maturity in this species. In this study, RNA-Seq and differential gene expression analysis were performed to investigate gene expression for floral initiation and development in crested wheatgrass. Three cDNA libraries were generated and sequenced to represent three successive growth stages by sampling leaves at the stem elongation stage, spikes at boot and anthesis stages. The sequencing generated 25,568,846; 25,144,688 and 25,714,194 qualified Illumina reads for the three successive stages, respectively. *De novo* assembly of all the reads generated 311,671 transcripts with a mean length of 487 bp, and 152,849 genes with an average sequence length of 669 bp. A total of 48,574 (31.8%) and 105,222 (68.8%) genes were annotated in the Swiss-Prot and NCBI non-redundant (nr) protein databases, respectively. Based on the Kyoto Encyclopedia of Genes and Genome (KEGG) pathway database, 9,723 annotated sequences were mapped onto 298 pathways, including plant circadian clock pathway. Specifically, 113 flowering time-associated genes, 123 *MADS-box* genes and 22 *CONSTANS-*LIKE (*COL*) genes were identified. A *COL* homolog DN52048-c0-g4 which was clustered with the flowering time genes *AtCO* and *OsHd1* in *Arabidopsis* (*Arabidopsis thaliana* L.) and rice (*Oryza sativa* L.), respectively, showed specific expression in leaves and could be a *CONSTANS* (*CO*) candidate gene. Taken together, this study has generated a new set of genomic resources for identifying and characterizing genes and pathways involved in floral transition and development in crested wheatgrass. These findings are significant for further understanding of the molecular basis for late maturity in this grass species.

## Introduction

The crested wheatgrass complex (*Agropyron*) plays an important role in the provision of forage for ruminant animals in temperate semi-arid regions of North America. This complex has approximately 15 species and is an important perennial genus of the tribe Triticeae [1]. There are three ploidy types in the genus: diploid (2n = 2x = 14), tetraploid (2n = 4x = 28), and hexaploid (2n = 6x = 42). In Canada, *Agropyron cristatum* L. is the most common species of crested wheatgrass, with both diploid and tetraploid cultivars grown. The tetraploid type is more popular than diploid type. It is recognized as a valuable grass for spring grazing and hay production. However, once crested wheatgrass reaches the stage of flowering, nutritive value declines rapidly and plants become less palatable to grazing animals. Thus, the development of late maturing cultivars is a goal for crested wheatgrass breeding programs. However, it is a challenge to manipulate the timing of its floral transition, as there is little understanding of flowering genes and their expression in this species.

Flowering is a complex trait controlled by multiple genes and has considerable impacts on the adaptability, biomass and economic value in agricultural crops. Many genes involved in various flowering pathways, e.g. photoperiod, vernalization and autonomous pathways, have been identified in the model plant *A*. *thaliana* [2,3]. The photoperiod pathway controls flowering time in response to seasonal changes in day length. Two photoperiod responsive genes, namely, *CONSTANS* (*CO*) and *FLOWERING LOCUS T* (*FT*), are vital in regulating photoperiodic flowering [3,4]. In *Arabidopsis*, GIGANTEA (GI) protein, which is the output from the circadian clock, activates the expression of *CO* gene. The role of CO protein in flowering is to activate the *FT* gene which promotes flowering by acting upstream of the floral meristem identity genes *APETALA 1* (*AP1*), *LEAFY* (*LFY*) and *CAULIFLOWER* (*CAL*) [4]. The CO/FT module appears to be conservative and genes with similarity to *GI*, *CO* and *FT* have been identified and characterized in several grass species [5–11]. Vernalization is the process by which prolonged exposure to cold renders plants competent to flower [12]. It prevents seeds sown in late summer or early fall from developing into flowering plants. In *Arabidopsis*, vernalization reduces the activity of central flowering repressor which is encoded by the *FLOWERING LOCUS C* (*FLC*) gene [13]. In cereals, three genes (*VERNALIZATION1* [*VRN1*], *VRN2* and *VRN3*) have been identified and are thought to form a regulatory loop to control the timing of flowering [14].

In contrast with those genes identified in important annual grass species like rice (*Oryza sativa* L.) and wheat (*Triticum aestivum* L.), almost no research has been done on genes regulating the vegetative-to-reproductive transition in crested wheatgrass. Crested wheatgrass is an obligate outcrossing species, and thus the high level of individual heterozygosity and population heterogeneity makes its genetic and molecular studies more difficult. Recently, next-generation deep-sequencing technologies such as Solexa/Illumina RNA-sequencing (RNA-Seq) have provided new approaches to study global transcriptome profiles for species without reference genomes. RNA-Seq can study transcripts of a certain trait in a given developmental stage and has been used to investigate the transcriptomic profiles in some grass species [15,16]. Zhang et al. [17] performed *de novo* transcriptome sequencing of tetraploid crested wheatgrass to develop genomic resources for wheat genetic improvement. However, little attention has been paid to identify and characterize genes for flowering in crested wheatgrass.

To enhance our understanding of the genetic control of flowering in crested wheatgrass, an RNA-Seq analysis was conducted of the flowering transcriptome over three developmental stages: stem elongation stage (VS), boot stage (BS) and anthesis stage (AS) in tetraploid crested wheatgrass. The specific objectives of this study were: (1) to perform *de novo* assembly of three different RNA-Seq libraries to develop genomic resources for flowering traits and (2) to conduct a differential gene expression analysis among the three stages to identify genes involved in specific pathways for floral transition. It was hoped that this analysis would not only provide some baseline information and genomic resources for identifying and characterizing genes associated with floral initiation and development in this species, but also allow for better understanding of the molecular mechanisms controlling plant flowering.

## Materials and methods

### Plant materials and growth conditions

Four tetraploid crested wheatgrass plants, one from each line (Plant Introduction No. PI598641, PI439914, W625134 or PI439914), with the same flowering date in 2015 and 2016 growing seasons (S1 File) were selected for RNA-Seq analysis. These plants were grown in the field plots at Agriculture and Agri-Food Canada Saskatoon Research Farm, Saskatoon, SK, Canada. The field plots were established in July of 2014 on 1 m centers. The samples of the four selected plants were collected at the same time as follows: leaf tissues at stem elongation stage (approximately E0), spikes at boot stage 7-d after the first sampling (approximately R0) and spikes at the anthesis (flowering) stage (R4), 30-d from the second sampling, respectively according to Moore et al., (1991) [18]. The collected tissues were immediately frozen and stored in liquid nitrogen for RNA extraction.

### RNA extraction, cDNA library construction and Illumina sequencing

For each plant, total RNA was extracted from approximately 100 mg raw material at the three developmental stages, respectively, using the Qiagen RNeasy Plant mini kit (Qiagen) according to the manufacturer’s protocol. RNA quantification was performed using Nanodrop 8000 (Thermo Fisher) and RNA integrity was assessed via the RNA 6000 Nano labchip on 2100 Agilent Bioanalyzer (Agilent Technologies). For each growth stage, 1.25 ug RNA from each plant was pooled and used for subsequent analysis. The pooled RNA samples were subsequently used in cDNA library construction. Three cDNA libraries were prepared using a TruSeq® RNA sample preparation Kit from Illumina (San Diego, CA, USA). Paired-end libraries were sequenced using the Illumina HiSeq® 2500 system at National Research Council, Saskatoon, SK, Canada. All raw sequences were deposited in the National Center for Biotechnology Information Short Read Archive under accession number SRP096782.

### Transcriptome *de novo* assembly

The raw sequence reads for three floral development stages from the image data output from the sequencing facility were quality checked by FASTQC (http://www.bioinformatics.babraham.ac.uk/projects/fastqc/). Based on the FASTQC results, a filtering was performed to remove ribosomal RNA (rRNA) contamination and trimming to remove low quality bases and adapters. SortMeRNA was used to identify rRNA in the raw reads [19]. Trimmomatic [20] was used to clean the raw reads by removing low-quality reads (Q value = 20 as threshold), partial adapter sequences and reads with ambiguous bases ‘N’, based on the setting of paired-end mode, phred33 and threads 6. *De novo* assembly of the crested wheatgrass transcriptome was accomplished following the online instructions of Trinity [21,22]. The quality of *de novo* transcriptome assembly, including the number, total bases, mean length, and N50 of transcripts and genes, was checked using perl script “TrinityStats.pl” of the Trinity pipeline. Corset [23] was used to cluster relevant transcripts into genes. The bam files used for Corset analysis were produced by multi-mapping the reads to the transcriptome using Bowtie2 [24]. Each sample had one bam file. The assembled transcripts and genes were analyzed with the perl script “fasta_seq_length.pl” of the Trinity pipeline to get the sequence length for each transcript and gene.

### Sequence annotation and classification

Blast2go (http://www.blast2go.com/b2ghome) was used to align genes against the National Centre of Biotechnology Information (NCBI) nr protein database for function annotation. Local blastx was performed to search gene sequences against the Swiss-Prot protein database. The e-value cutoff was set at 1e^-5^. Gene name was assigned to each gene based on top Blastx hit with the highest score. The genes related to flowering time and floral development were explored based on the gene names. Similarity distribution and species distribution analysis was based on annotation from Blast2go. For each annotated transcript, the top blastx hit was used for analysis. The annotated sequences were searched in the KEGG Automatic Annotation Server (KAAS) [25,26] using the bi-directional best hit method. Specific metabolic pathways were identified from the output in KAAS.

### Phylogenetic tree

Multiple protein alignments were performed using ClustalW (http://www.ebi.ac.uk/clustalw/) based on the AtCO and OsHd from *A*. *thaliana* and rice, respectively, as well as CO homologs TaHd1-1 and TaHd1-3 from wheat and HvCO1 and HvCO2 from barley (*Hordeum vulgare* L.), along with the putative CO homologs in crested wheatgrass with CCT domain. The following 22 protein sequences were obtained and aligned for phylogenetic analysis: AtCO (NP_197088), OsHd1 (ABB17664), TaHd1-1 (BAC92735), TaHd1-3(BAC92736), HvCO1(AF490467_1), HvCO2(AF490470_1), AtCOL1 (NP_197089), AtCOL2 (NP_186887), AtCOL3 (Q9SK53), AtCOL4 (Q940T9), AtCOL5 (Q9FHH8), AtCOL6(Q8LG76), AtCOL7 (Q9C9A9), AtCOL8 (Q9M9B3), AtCOL9 (NP_001118599), AtCOL10 (Q9LUA9), AtCOL11 (O23379), AtCOL12 (Q9LJ44), AtCOL13 (O82256), AtCOL14 (O22800), AtCOL15 (Q9C7E8) and AtCOL16 (Q8RWD0). MEGA software (version 6.0) [27] was used to construct a phylogenetic tree with the aligned protein sequences. The neighbor-joining method was used with pairwise deletion option, Poisson correction model, and the 1000 bootstrap replicates.

### Differential gene expression analysis

The alignment-based qualification method RSEM [28] was used to estimate gene abundance. The *de novo* assembly of three RNA-Seq libraries was used as reference. Each RNA-Seq library was separately aligned to the reference, using Bowtie [29]. The differentially expressed genes (DEGs) were analyzed using R Bioconductor package, edgeR [30]. The edgeR dispersion value was explored between 0.1 and 0.4, and 0.2 was adopted based on the number of DEGs. The threshold of the P-value in multiple tests was determined by the value for the false discovery rate (FDR) [31]. The threshold to judge the significance of gene expression differences was ‘‘FDR ≤ 0.001 and the absolute value of Log_2_ fold change (Log_2_^FC^) ≥ 4”. Local blastx was performed to search DEGs against the KEGG database. A tabular BLAST output from the local blastx was analyzed by the KEGG Orthology Based Annotation System (KOBAS) program [32] for pathway enrichment analysis. Significantly enriched pathways were defined by taking a corrected P-value = 0.05 as the threshold.

To assess the accuracy of DEG detection, we also performed a quantitative real-time PCR (qRT-PCR) validation of DEGs related to flowering time and floral development using two plants randomly selected from the Plant Introduction No.W625134 and 439914, respectively. Specifically, total RNA at the VS and BS stages of these two plants was extracted, respectively. The RNA extraction method was the same as described for the RNA-Seq library preparation and sequencing. Extracted RNA was treated with DNAse I (Ambion), and cDNA was synthesized using the SuperScript® First-Strand Synthesis System for RT- PCR (invitrogen) according to the manufacturer’s instruction. The sequences of the specific primer sets for each tested gene were designed using the PrimerQuest Tool (https://www.idtdna.com/Primerquest/Home/Index) and listed in S2 File. The glyceraldehyde-3-phosphate dehydrogenase (*GAPDH*) gene of crested wheatgrass (DN67262-c0-g1) was used as an internal control for normalization. Three separate first-strand cDNA reactions were analyzed in duplicate for each sample. The qPCR analysis was performed with SsoFast EvaGreen supermix (Bio-rad) according to the manufacturer’s instructions using a Bio-rad CFX96^TM^ system.

## Results

### Illumina sequencing and sequence assembly

**Fig 1:**
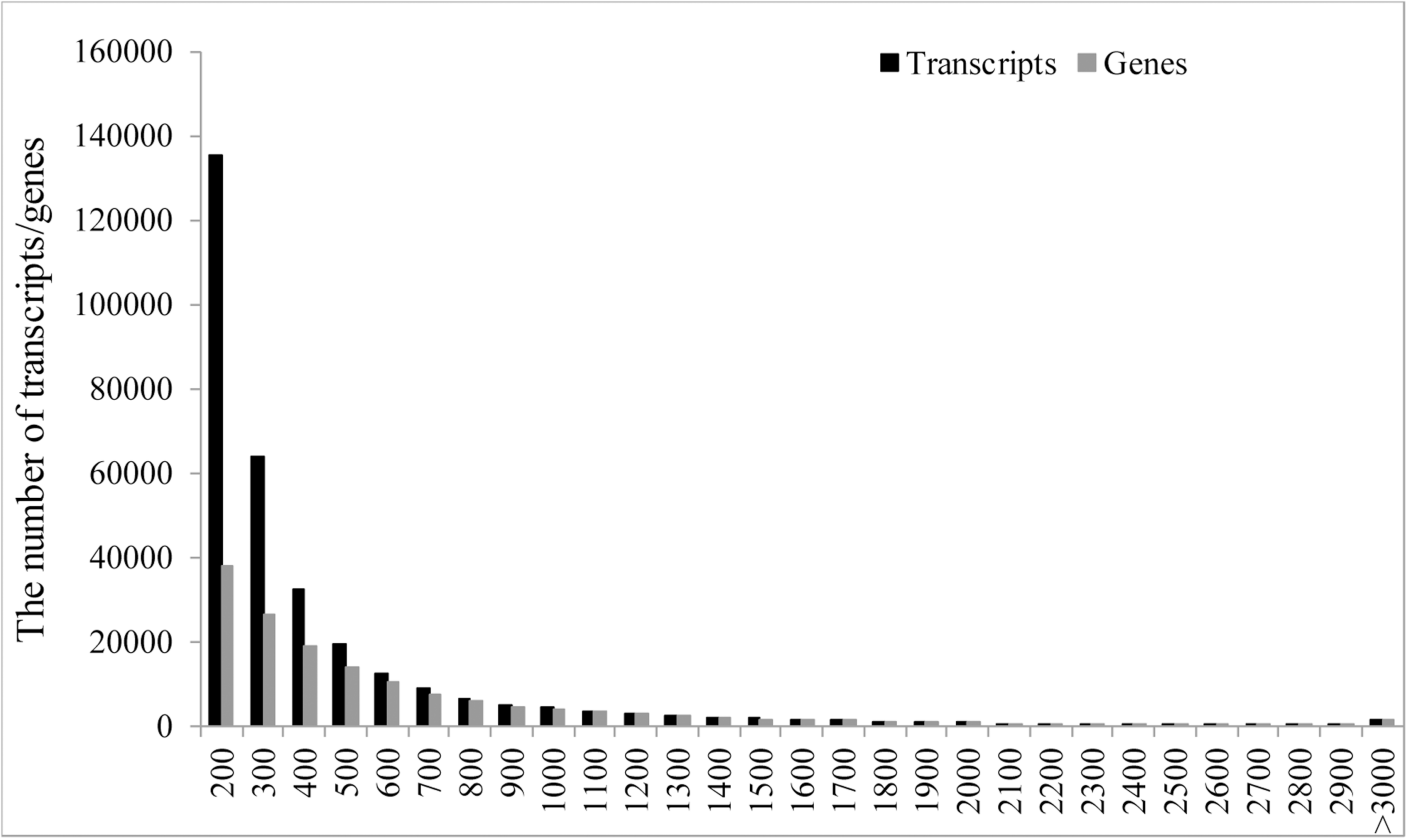
Sequence length distributions of transcripts/genes assembled from Illumina sequence reads from three floral development stages of crested wheatgrass.

Illumina HiSeq® 2500 paired-end sequencing generated 25,568,846; 25,144,688 and 25,714,194 qualified sequence reads for VS, BS and AS stages, respectively (Table 1). *De novo* assembly of 69,484,618 clean reads with mean length of 122 bp generated 311,671 transcripts with a total of 151,835,903 bases (Table 1, S3 File). The average length of the transcripts was 487 bp and N50 of the transcripts was 558 bp. The sequence length distributions of the transcripts and genes were shown in Fig 1. Using paired-end reads, these transcripts were further clustered into 152,849 genes by Corset, with a mean length of 669 bp. A summary of sequence reads, assembled transcripts and genes for each library are presented in Table 1.

**Table 1:** Summary of Illumina transcriptome sequencing from three floral development stages of crested wheatgrass: Stem elongation stage (VS), boot stage (BS) and anthesis stage (AS).

Stage	VS	BS	AS	All stages
Raw reads	25,568,846	25,144,688	25,714,194	76,427,728
Clean reads	22,713,895	23,585,737	23,184,986	69,484,618
Total clean nucleotides (nt)	2,779,828,358	2,902,969,649	2,821,635,887	8,504,433,894
Average read length	122	123	122	122
Total Trinity transcripts	128,690	200,412	114,590	311,671
Mean length of contigs	491	508	470	487
N50 of contigs	580	604	534	558
Total assembled bases	63,237,556	101,928,935	53,813,990	151,835,903
Total genes	87,076	118,724	70,698	152,849
Mean length of genes	781	742	863	669
N50 of genes	1134	1006	1273	883
Total assembled bases	68,035,269	88,055,369	61,029,401	102,318,880

### Sequence annotation

**Fig 2:**
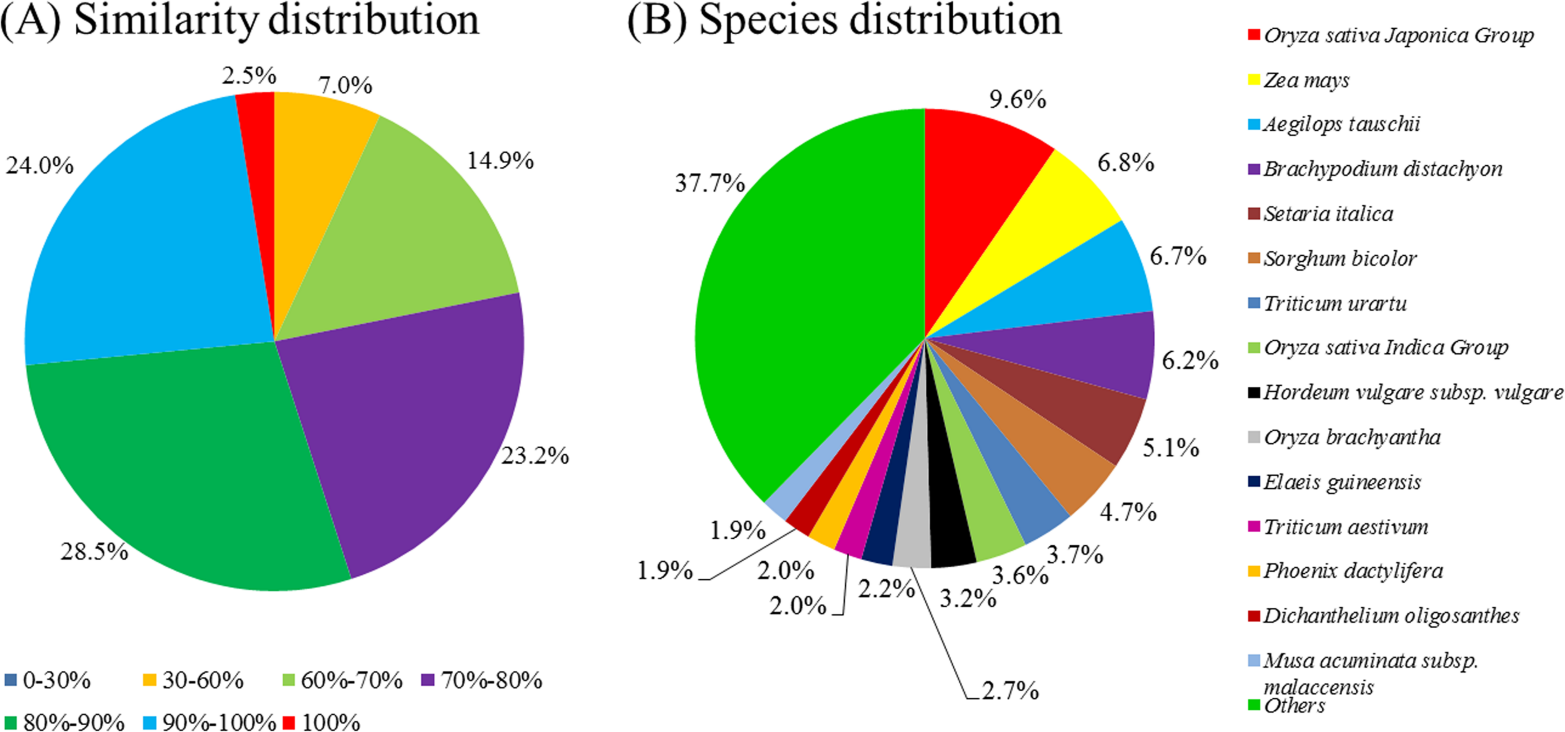
Statistics of sequence-homology search against NCBI nr protein database with respect to similarity (A) and species (B).

Assembled genes were annotated using Blastx program with an E-value threshold of 10^−5^ against Swiss-Prot and NCBI non-redundant (nr) protein databases. A total of 48,574 (31.8%) and 105,222 (68.8%) genes showed significant similarity to known proteins in the Swiss-Prot and NCBI nr protein databases, respectively. The sequences and annotation of the 105,222 genes were presented in S4 and S5 Files, respectively. The annotation information from nr database was used for similarity distribution and species distribution analysis. The similarity distribution showed that 55.0% of the matches were of high similarity ranging from 80% to 100% similarity as reported in the Blastx results whilst 38.1% of the matches were of similarity ranging from 60% to 80% (Fig 2A). Further analysis of the matching sequences indicated that 9.6% of the sequences showed the closest matches with sequences from *Oryza sativa* while 6.8% and 6.7% of the sequences showed closest matches with sequences from *Zea mays* and *Aegilops tauschii*, respectively (Fig 2B).

### Flowering time-associated genes and *MADS-box* genes

We obtained 113 genes associated with flowering time (S6 File) based on the annotations of assembled genes. The number of flowering time homologous genes in the crested wheatgrass transcriptome data is summarized in Table 2. These include photoreceptor genes cryptochromes (*CRY1* and *CRY2*) and photochromes genes (*PHYA* and *PHYC*); photoperiod pathway genes *EARLY FLOWERING 3* (*ELF3*), *PHYTOCHROME INTERACTING FACTOR 3* (*PIF3*), *CIRCADIANCLOCK-ASSOCIATED1* (*CCA1*), *LATE ELONGATED HYPOCOTYL* (*LHY*), *TIMING OF CAB* (*TOC1*), *GI*, *CHALCONE SYNTHASE* (*CHS*) and *CO*; vernalization pathway genes such as *VRN1*, *VERNALIZATION INDEPENDENCE 4* (*VIN4*) and *VERNALIZATION INSENSITIVE 3* (*VIN3*); floral integrator pathway genes *FT*, *APETALA 2* (*AP2*) and *SUPPRESSOR OF OVEREXPRESSION OF CONSTANS1* (*SOC1*) and floral meristem identity genes *LFY*. Also, *MADS-box* genes are important resources for the study of floral organ formation and 123 *MADS-box* genes were identified (S7 File). Moreover, three chromatin-related flowering time genes *EMBRYONIC FLOWER 2* (*EMF2*), *FERTILIZATION-INDEPENDENT ENDOSPERM* (*FIE*) and *SWITCH/SUCROSE NONFERMENTING* (*SWI*) were detected.

**Table 2:** Identification of 113 flowering time-associated genes from crested wheatgrass transcriptome based on NCBI nr database annotation.

Category	Homologous gene in the nr database	The number of corresponding genes in our transcriptome data
Photoreceptor	*PHYA*	4
	*PHYC*	3
	*CRY1*	3
	*CRY2*	1
	*ELF3*	4
	*PIF3*	5
Circadian clock	*TOC1*	1
	*CCA1/LHY*	1
	*GI*	2
	*CHS*	29
	*CO*	22
Vernalization pathway	*VRN 1*	1
	*VIN 4*	1
	*VIN 3*	4
Floral integrator pathway	*FT*	21
	*AP2*	3
	*SOC1*	1
Chromatin related	*EMF2*	4
	*FIE*	1
	*SWI*	1
Floral meristem identity	*LFY*	1

### Metabolic pathway assignment by KEGG

**Fig 3:**
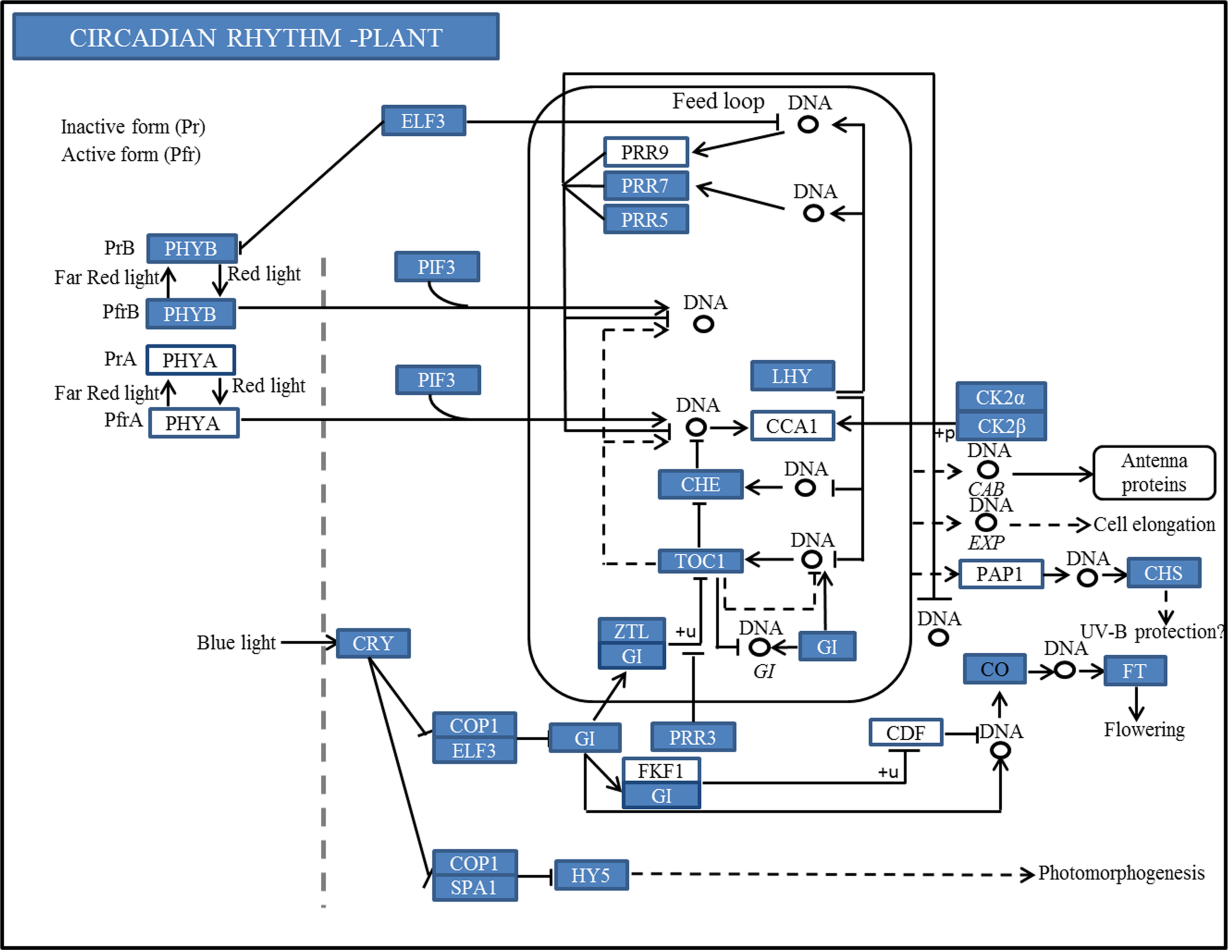
The plant circadian rhythm pathway revealed by KEGG annotation in three floral development stages of crested wheatgrass. The genes highlighted in blue are found in our transcriptome data.

Mapping 105,222 annotated genes to the reference canonical pathways in KEGG revealed a total of 9,723 genes that were assigned to the 298 KEGG pathways (S8 File). The pathways with the most represented genes were the metabolism pathway (830 genes) and the biosynthesis of secondary metabolites (384 genes). Interestingly, a circadian rhythm pathway involving 21 genes was also found in KEGG, and the detailed metabolic pathway for the circadian rhythm is shown in Fig 3. Circadian rhythm is an important part of the photoperiod pathway for plant flowering. In *A*. *thaliana*, the circadian oscillator, which is at the core of photoperiod pathway, is composed of the interlocked feedback loop formed by a pseudo response regulator (PRR) and major transcriptional factors CCA1, LHY and TOC1. The morning-expressed CCA1/LHY suppresses TOC1 expression by binding to its promoter. Moreover, CCA1/LHY activates the expression of PRR7/9 in the morning and then PRR7/9 represses the transcription of CCA1/LHY during the rest of the day. By contrast, the evening-expressed TOC1 activates the expression of CCA1/LHY. In the flowering transcriptome of crested wheatgrass, not only the homologs of these major factors *CCA1*, *LHY*, *TOC1* and *PRR7*, but also photoperiod genes *ELF3*, *PIF3*, *GI*, *CO* and *FT* were mapped to the circadian rhythm pathway. The pathway identified here not only confirms that the photoperiod pathway is involved in flowering in crested wheatgrass, but also provides valuable resource for investigating the photoperiod pathway and flowering-related processes in crested wheatgrass.

### Identification of crested wheatgrass *CONSTANS*-like (*COL*) gene family

**Fig 4:**
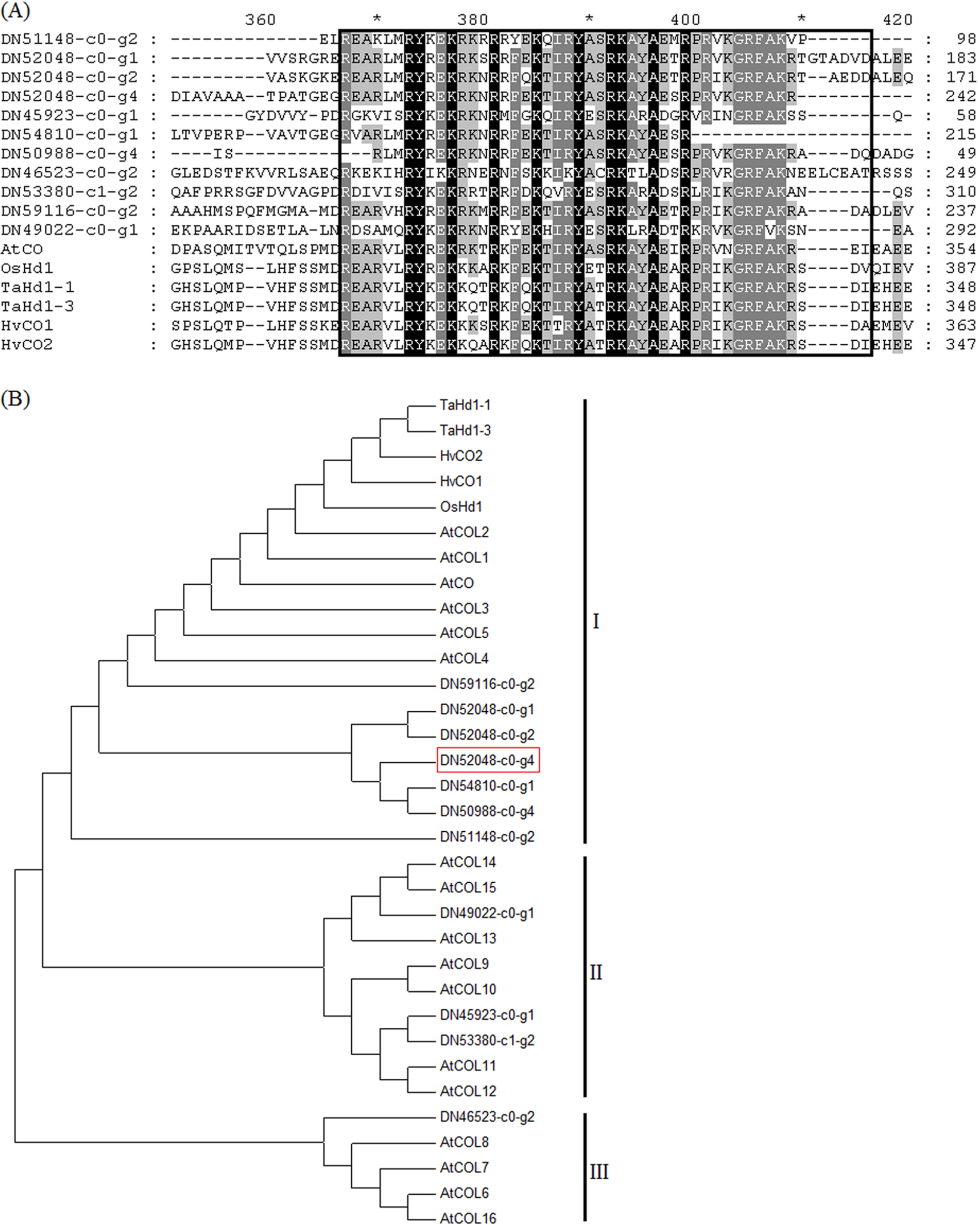
Characterization of the putative CONSTAN-like proteins in crested wheatgrass.

In *A*. *thaliana*, *CO* gene plays a central role in the photoperiodic control of flowering. One CO and 16 CO-like proteins named AtCOL1-AtCOL16 formed a gene family of 17 members in *A*. *thaliana*. All members of this gene family contain one or two B-box domains at the N-terminus and a CCT domain at the C-terminus [33,34]. A total of 22 homologs of the *COL* genes were identified in our assembled transcriptome database (Table 2 and S6 File). Amino acid sequence alignments indicated that 11 putative proteins of these homologs in crested wheatgrass have conserved CCT domains (Fig 4A). The phylogenetic analysis of these putative COL homologs with COL proteins in *A*. *thaliana*, rice, wheat and barley revealed that all members of homologs with conserved CCT domains in crested wheatgrass could be divided into three divergent groups (Fig 4B). Seven COL homologs (DN59116-c0-g2, DN52048-c0-g1, DN52048-c0-g2, DN54810-c0-g1, DN52048-c0-g4, DN50988-c0-g4 and DN51148-c0-g2) were assigned in group I and were clustered with AtCOL3/AtCOL4/AtCOL5. They were closely related to AtCO and OsHd1. DN49022-c0-g1, DN45923-c0-g1 and DN53380-c0-g2 were closely related to AtCOL9 and AtCOL10 and clustered in group II. DN46523-c0-g2 was clustered with AtCOL6 and AtCOL16 in group III. These results suggested that the COL proteins have evolved before the divergence of the monocots and dicots [7,35,36].

### Differential gene expression during floral initiation and development

**Fig 5:**
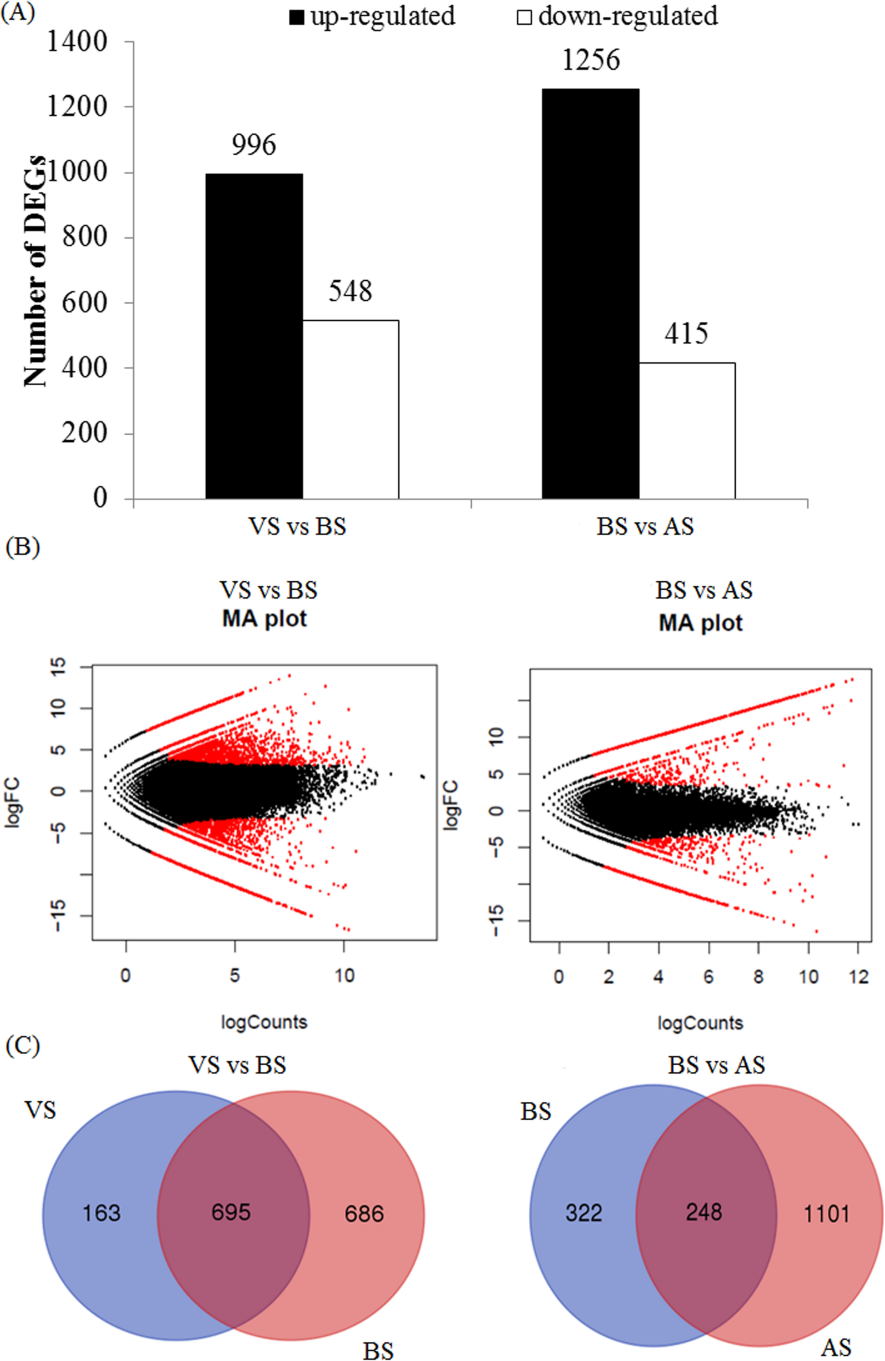
Differential expressed genes (DEGs) in two pairs of the floral developmental stages of crested wheatgrass [stem elongation stage (VS) vs. boot stage (BS) and boot stage (BS) vs. anthesis stage (AS)].

Putative homologs of *COL* and some other important genes involved in controlling flowering time in crested wheatgrass have been identified. To better understand the molecular mechanisms that regulate the genetic pathways of floral transition and floral development, a genome-wide expression analysis was performed for the VS, BS and AS developmental stages. The expression of each gene was calculated using the numbers of reads mapping onto the assembled transcriptome. Comparisons of gene expression showed that a total of 1544 genes, including 996 up-regulated and 548 down-regulated genes were identified in BS when compared to VS (Fig 5A). An overview of the expression patterns for VS vs BS was shown in Fig 5B. The expression changes of 1544 differentially expressed genes (DEGs) ranged from 17-fold to -14-fold. There were 163 genes showing specific expression in VS while 686 genes showing specific expression in BS (Fig 5C, S9 File). KOBAS was used to further identify biosynthetic pathways and to explore the functions of the DEGs in detail. Some of DEGs, which were up-regulated in the BS, were significantly enriched to the pathway of steroid biosynthesis by KOBAS (S10 File).

Comparing gene expressions between BS and AS revealed that 1671 DEGs were identified in AS when compared to BS, including 1256 up-regulated and 415 down-regulated genes (Fig 5A). A corresponding view of the expression patterns for BS vs AS was depicted in Fig 5B. The changes of 1671 DEGs ranged from a 17-fold to an -18-fold. Among these genes, 322 genes were specifically expressed in BS while 1101 genes showed specific expression in AS (Fig 5C, S9 File). In KOBAS enrichment, 13 gene sets were significantly enriched and they are related to ribosome, lysosome, starch and sucrose metabolism, fatty acid metabolism, cutin, suberine and wax biosynthesis. Most of these DEGs were up-regulated in the AS stage (S10 File).

DEGs related to flowering time and floral development were also identified. The flowering time-associated genes showed higher expression levels in VS than that in BS, and the functional annotation for these genes was listed in Table 3. DN61589-c0-g2, the homologous gene to the plant circadian clock gene *LHY*, was expressed 11 times higher in VS than that in BS. DN59278-c0-g2, the homologous gene of plant circadian clock gene *GI*, was expressed 5 times higher in VS. The *CO*/*FT* regulatory system was found to be associated with flowering initiation in crested wheatgrass. DN52048-c0-g4, which is the homologous gene of *CO* expressed about 5 times higher in VS. DN56531-c3-g1 is the homologous gene for *FT* and its expression elevated about 8 times in VS. DN53048-c0-g2, homologous gene of *flowering-promoting factor 15* also had 7 times elevation in VS compared to that in BS. DN53956-c0-g1, the homologous gene of *ZCN26*, a member of the maize *FT*-related gene family, showed about 10 times higher expression level in VS. These results indicated that photoperiod pathway is involved in flower initiation in crested wheatgrass. Genes involved in floral development were expressed significantly higher in BS (Table 4). These up-regulated genes in BS were involved in flower organ formation and floral development. DN56706-c0-g5, DN60175-c2-g2, DN56706-c0-g6 and DN36907-c0-g1, the homologous genes of *MADS-box* transcription factors, were expressed from 5 to 12 times higher in BS than VS and were correlated with floral development. DN55848-c0-g1, a homologous gene of auxin response factor, showed 10 times higher expression in BS. These identified genes and their sequences are useful for future functional studies on floral development in crested wheatgrass.

**Table 3:** List of 13 differentially expressed genes related to flowering time of crested wheatgrass between stem elongation stage (VS) and boot stage (BS).

			VS vs BS	
Gene ID	Putative function	Nr ID^a^	log_2_FC^b^	FDR^c^
DN61589-c0-g2	LHY isoform X1 [*Oryza sativa Japonica* Group]	AFO69281.1	10.8	4.46E-10
DN43771-c0-g1	heme oxygenase 1	ADG56719.1	10.7	6.76E-10
DN60312-c2-g3	MADS box VRT- partial	CAM59070.1	10.4	4.94E-09
DN53956-c0-g1	ZCN26 [*Zea mays*]	BAK02662.1	9.7	1.93E-07
DN60312-c2-g5	MADS box VRT- partial	ADR51708.1	9.7	2.08E-07
DN54250-c0-g1	WD repeat-containing 70	EMS64159.1	9.6	2.43E-07
DN59116-c0-g2	zinc finger CONSTANS-LIKE 2-like	EMS63149.1	8.6	3.98E-05
DN60312-c2-g2	MADS box VRT-2 [*Hordeum vulgare*]	CAM59070.1	8.3	1.98E-10
DN55669-c2-g1	MADS-domain transcription factor [*Zea mays*]	ADR51708.1	8.3	0.0002
DN56531-c3-g1	flowering locus T 1 [*Hordeum vulgare*]	CAE53888.1	8.2	0.0002
DN53048-c0-g2	flowering-promoting factor 15	EMT26987.1	7.2	8.14E-11
DN59278-c0-g2	GIGANTEA [*Oryza sativa Japonica* Group]	CDM81775.1	4.8	7.06E-08
DN52048-c0-g4	CONSTANS CO6 [*Zea mays*]	BAJ98422.1	4.5	0.0001

^a^ nr ID is the protein accession number in NCBI non redundant protein database

^b^ logFC stands for logFoldChange, where it is log base 2

^c^ FDR stands for false discovery rate, which is used to determine the threshold P value in multiple tests

**Table 4:** List of 20 differentially expressed genes related to floral development of crested wheatgrass between stem elongation stage (VS) and boot stage (BS).

			VS vs BS	
Gene ID	Putative function	Nr ID^a^	log_2_FC^b^	FDR^c^
DN61161-c0-g1	MCM complex family [*Zea mays*]	BAJ99818.1	-12.5	2.52E-07
DN56706-c0-g5	MADS-box transcription factor 8	AAQ11687.1	-12.5	2.69E-07
DN60175-c2-g2	MADS-box transcription factor 3 isoform X1	ALM58836.1	-11.8	1.66E-06
DN61626-c1-g1	kinesin KIF22 [*Oryza brachyantha*]	EMT04655.1	-11	1.02E-05
DN55924-c2-g1	glycerol-3-phosphate 2-O-acyltransferase 6	AKL71379.1	-11	1.13E-05
DN52987-c0-g1	DL related [*Zea mays*]	BAJ54068.1	-10.6	2.93E-05
DN55370-c0-g1	PISTILLATA-like MADS-box transcription partial	AMO12834.1	-10.5	4.33E-05
DN53410-c0-g1	lysine-specific demethylase JMJ706-like isoform X1	BAK00861.1	-10.2	7.40E-05
DN55848-c0-g1	auxin response factor	AEV40985.1	-10.2	7.93E-05
DN58910-c2-g4	*leafy* hull sterile partial	EMS65447.1	-9.9	0.0002
DN43571-c0-g1	WD repeat-containing 61	BAK04016.1	-9.9	0.0002
DN54871-c3-g1	WW domain-binding 11-like	XP_003557433.1	-9.5	0.0004
DN62455-c0-g1	S-acyltransferase 21 isoform X1	BAJ88912.1	-9.4	0.0005
DN62601-c2-g1	polycomb group EMBRYONIC FLOWER 2 isoform	BAJ97315.1	-9.3	0.0007
DN62521-c1-g11	probable chromo domain-containing LHP1	BAJ95567.1	-9.2	0.0008
DN59231-c1-g2	DNA polymerase alpha subunit B	BAK05466.1	-9.2	0.0009
DN56706-c0-g6	MADS-box transcription factor 7	BAF75017.1	-8.5	5.18E-07
DN56923-c0-g1	DNA replication licensing factor mcm2	AAS68103.1	-7.2	1.24E-05
DN57261-c0-g1	systemin receptor SR160	CDM83621.1	-5.4	0.0006
DN36907-c0-g1	MADS-box transcription factor 58	CAM59041.1	-5.1	0.001

^a^ nr ID is the protein accession number in NCBI non redundant protein database

^b^ logFC stands for logFoldChange, where it is log base 2

^c^ FDR stands for false discovery rate, which is used to determine the threshold P value in multiple tests

**Fig 6:**
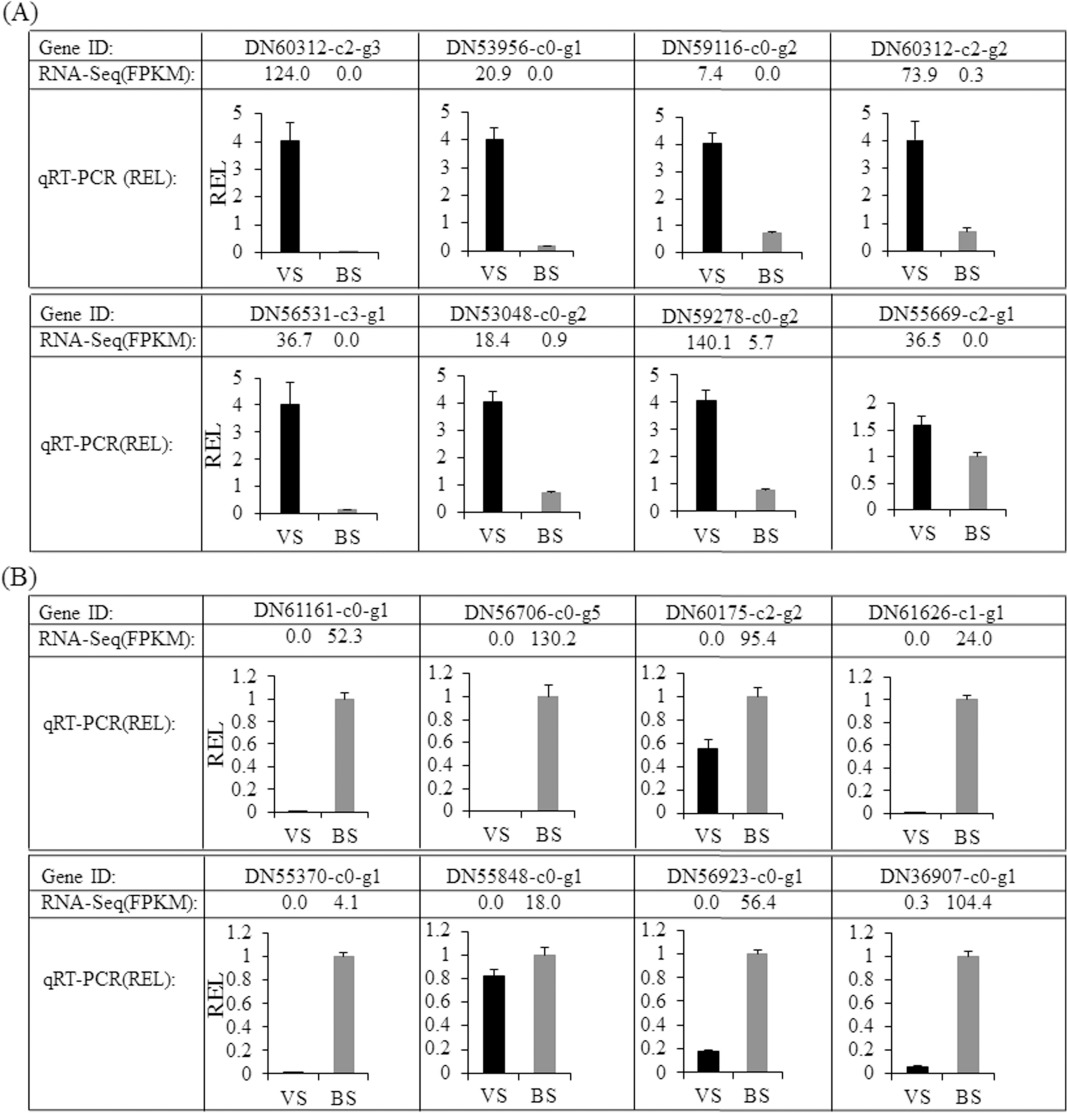
The qRT-PCR analysis of gene expression in the floral developmental stages of crested wheatgrass [stem elongation stage (VS) vs. boot stage (BS)].

Our qRT-PCR analysis of eight flowering time-associated genes randomly selected from Table 3 showed up-regulation at the stem elongation stage (Fig 6A). Similarly, eight flowering development genes randomly selected from Table 4 showed higher expression at the boot stage (Fig 6B). These qRT-PCR results helped to confirm the reliability of our differential gene expression analysis.

## Discussion

This study represents the first attempt to use RNA-Seq to analyze the transcriptome of different floral developmental stages in crested wheatgrass. The analysis revealed several interesting findings. First, there were 311,671 transcripts identified with a mean length of 487 bp and 68.8% of 152,849 genes were annotated in the Swiss-Prot and NCBI nr protein databases, including the identification of 113 flowering time-associated genes, 123 *MADS-box* genes and 22 *COL* genes. Second, 9,722 annotated genes were mapped onto 298 pathways using the KEGG pathway database, which including plant circadian rhythm. Third, a *COL* homolog DN52048-c0-g4 seemed to be a *CO* candidate gene in crested wheatgrass. These findings not only demonstrated the effectiveness of RNA-Seq and differential gene expression analysis in the identification and characterization of genes related to complex traits in a species without a reference genome, but also advanced our understanding of the conserved photoperiod-circadian clock-CO-FT regulatory circuit for flowering initiation in crested wheatgrass.

We generated 69 million high quality sequence reads from three cDNA libraries representing the transition from juvenile to the adult phase and identified 311,671 transcripts. These transcripts were clustered into 152,849 genes and 68.8% of genes were annotated in the NCBI nr protein database. The proportion of annotated genes was compatible with the report of 62.4% of 73,664 genes detected from a similar RNA-Seq analysis of flag leaf and young spike samples in tetraploid crested wheatgrass plants [17]. However, we do not know the reason(s) for such a considerable proportion of un-annotated genes obtained from the two similar studies. Some un-annotated genes may represent novel genes associated with vegetative and reproductive growth of crested wheatgrass in unique processes and pathways. It is possible that those un-annotated genes detected from the tetraploid crested wheatgrass may contain many separate paralogs, chimeras and differentially expressed isoforms. Also, the stringent conditions used for running Bowtie2 to map reads to the transcriptome for gene detection using Corset would generate more genes with paralogs for lowering gene annotation.

Many genes associated with vernalization and photoperiod pathways for floral transition have been identified in crested wheatgrass (Table 2 and S6 File). Vernalization is the process by which flowering is promoted by prolonged exposure to cold period. Vernalization removes blocking of flowering so that the plant can perceive inductive signals, such as long-day photoperiod. The *FLC* gene of *A*. *thaliana* has been identified as a central flowering repressor which can directly interfere with *FT* expression in leaves [37]. In winter wheat and barley, three genes *VRN1*, *VRN2* and *VRN3* (*FT*) form a regulatory loop that regulates the initiation of flowering [38–41]. *VRN1* encodes an AP1-related MADS-box protein [42]. VRN2 has CCT zinc finger, functioning as floral repressor FLC [43–46]. *VRN3* corresponds to an ortholog of *A*. *thaliana FT* [47]. During growth of vernalization-requiring cereals in the fall, *VRN2* represses *FT* to prevent flowering and *VRN1* is transcribed at very low levels [41,47,48,49]. However, when winter comes, *VRN1* expression level increases, which causing the repression of *VRN2* and activation of *VRN3* [41,49,50]. In our transcriptome data, homologs of *VRN1* and *FT* were found. However, no *VRN2* homologous gene has been detected. This may reflect the late sampling of tissue in our study and the high level of *VRN1* after vernalization repressed the expression of the *VRN2*. Also, two other vernalization genes homologous to *VIN3* and *VIN4* were found. As a cool-season grass, crested wheatgrass requires vernalization to induce floral initiation. These newly detected genes will allow us to further study the molecular mechanism of vernalization in this perennial species.

Photoperiod is an important factors regulating flowering [51]. The photoperiod pathway consists of three parts: photoreceptors, a circadian clock and an output pathway from the clock specific to flowering. Interactions between photoreceptors and the circadian clock are thought to allow plants to distinguish different day lengths. Phytochromes (PHYA-PHYE) detect red/far-red light and the cryptochromes (CRYs) detect blue light [52–55]. In our transcriptome data, homologous genes for phytochromes (*PHYA* and *PHYC*) and two cryptochromes (*CRY1* and *CRY2*) were found (Table 2 and S6 File). These photoreceptors process physical signals and produce a circadian clock. The circadian clock is an endogenous timekeeper that controls many rhythmic processes in organisms as they experience 24h cycle of day and night [56]. There are several genes in the long-day flowering pathway affecting both circadian rhythms and flowering time in *A*. *thaliana*. The *ELF3* gene is responsible for light input signals to the clock, measures daylength and represses flowering under short days [57]. The *elf3* mutant shows early, daylength-insensitive flowering [57]. The best-characterized transcriptional-translational regulatory feedback loop in the circadian clock is comprised of two morning-expressed Myb transcription factors, *CCA1* and *LHY*, and an evening-expressed gene *TOC1* [58,59]. This central loop interlocks with the morning and evening loops, forming the basic architecture of the plant circadian clock. Overexpression of either of *LHY* and *CCA1* resulted in the late flowering plants and the expression of several genes controlled by circadian clock was disrupted [59,60]. The mutation of *TOC1* can accelerate circadian rhythms and cause early flowering *A*. *thaliana* under short days [61]. The identification of these homologous genes (Table 2 and S6 File) confirmed that photoreceptors and circadian clock are conserved for flowering in crested wheatgrass.

The circadian clock controls the *CO* expression level rhythm through GI, a large plant-specific protein [62,63]. GI activates expression of CO protein, a B-box-type zinc finger transcription factor-encoding gene expressed in leaves [64,65]. When it coincides with a light period, indicating long days of summer, translated CO protein is stable and directly activates its prime target, the *FT* gene [64]. FT protein then acts as a transcription factor to activate expression of *APETALA1* (*AP1*) and promote floral meristem identity gene at the shoot apex [66,67]. Expression of *CO* in dark periods of a long night, however, results in degradation of CO protein and no FT activation [68]. *COL* genes have been identified in some plant species, each of which seems to have a large family with several genes [69]. There are 17 *COL* genes in *A*. *thaliana* [34] and at least 16 *COL* genes in rice [69]. In this study, eleven of the 22 crested wheatgrass COL homologs were identified with the conserved CCT domain. The phylogenetic analysis revealed that these COL proteins were classified into three conserved COL subgroups as defined previously [33,34,69]. COL proteins of different subgroups are expected to perform distinct biological function and our 11 COL proteins are expected to perform different biological roles. CO homolog DN52048-c0-g4 clustered with AtCO and OsHd1 in class I of the *COL* gene family. The *AtCO* gene integrates inductive photoperiod information via the circadian clock and actives the *FT* gene promoting flowering in *Arabidopsis*. *OsHd1* was found to encode an ortholog of the *AtCO* gene in rice [5]. Besides *CO* homologous genes, sequence homologs for *GI* and *FT* genes were also found in our transcriptome data, which suggests that the output pathway of circadian clock, CO and FT proteins all are involved in flowering in crested wheatgrass.

As crested wheatgrass is an open pollinated species, each plant is genetically heterogeneous and our bulk of four genotypes should be informative to our current DEG analysis with edgeR. Our effort has detected 1544 DEGs between VS and BS stages and 1671 genes between BS and AS stages. The circadian clock output gene *GI* (DN59278-c0-g2) showed higher expression in VS than that in BS, indicating its role in the transition to flowering. *CO* (DN52048-c0-g4) and *FT* (DN56531-c3-g1) homologs showed specific expressions in the leaves (VS) of crested wheatgrass. *CO* was reported to be expressed in the vasculature of *A*. *thaliana* leaves, and its role in flowering is to activate the expression of *FT* [70]. In both rice and *Arabidopsis*, FT is a strong promoter of flowering that is translocated from the vasculature of leaves to the shoot apical meristem [3,71]. Our phylogenetic data also showed that CO homolog (DN52048-c0-g4) clustered with AtCO and OsHd1, indicating that it could be the *CO* candidate gene responsible for integrating photoperiod and activating *FT* gene in crested wheatgrass. Thus, our DEG analysis was informative and provided additional support that ancient and evolutionarily adaptable module CO/FT is conserved in crested wheatgrass.

Altogether, our RNA-Seq analysis has generated a new set of genomic resources for characterizing genes and pathways involved in floral transition and development in crested wheatgrass. These genomic resources can be explored and utilized to develop marker-based tools for development of late maturing lines in this species. Our research findings clearly showed that vernalization and photoperiod pathways are involved in the regulation of flowering time in crested wheatgrass, and thus advanced our understanding of the mechanisms for flowering initiation in this species. Also, the photoperiod-circadian clock-CO-FT regulatory circuit is conserved in crested wheatgrass. However, many questions remain, like how GI is regulated by circadian clock and how it controls the expression of CO. The possible role of CO and FT in photoperiod response and whether FT moves from leaves to meristem as mobile flowering signals are worthy to pursue. The answers to these questions require more extensive expression analysis and successful cloning of these genes. Also, the research outputs presented here have clearly demonstrated the effectiveness of RNA-Seq and differential gene expression analysis in the identification and characterization of genes related to specific traits in species without a reference genome. These technologies have made the informative genomic investigation of a specific complex pathway such as floral development in a non-model plant practically possible.

## Supporting information

S1 File The flowering date of the four selected tetraploid accessions of crested wheatgrass in year 2015 and 2016 at Saskatoon, SK, Canada. (DOCX)

S2 File Primer pairs used for qRT-PCR analysis. (DOCX)

S3 File Sequences of 311,671 assembled transcripts in fasta format. (ZIP)

S4 File Sequences of 105,222 annotated genes in fasta format. (ZIP)

S5 File The annotation information for 105,222 annotated genes in text file. (ZIP)

S6 File A list of the 113 genes identified in the crested wheatgrass transcriptome showing homology with previously identified flowering time genes. (XLSX)

S7 File A list of the 123 *MADS-box* genes identified in the crested wheatgrass flowering initiation and development transcriptome in excel file. (XLSX)

S8 File A summary of 298 KEGG pathways identified in the crested wheatgrass flowering initiation and development transcriptome. (DOCX)

S9 File A list of genes showing specific expression in VS, BS and AS, respectively, in excel file. (XLSX)

S10 File A list of 14 statistically enriched pathways identified in differentially expressed genes using KOBAS database in excel file. (XLSX)

## References

[1] Dewey DR. The genomic system of classification as a guide to intergeneric hybridization with the perennial Triticeae In: Gustafson JP, editor. Gene manipulation in plant improvement. Springer; 1984 pp. 209–79.

[2] Amasino R. Seasonal and developmental timing of flowering. Plant J. 2010;61(6):1001–13. doi: 10.1111/j.1365-313X.2010.04148.x 20409274 10.1111/j.1365-313X.2010.04148.x

[3] Corbesier L, Vincent C, Jang S, Fornara F, Fan Q, Searle I, et al FT protein movement contributes to long-distance signaling in floral induction of *Arabidopsis*. Science. 2007;316(5827):1030–3. doi: 10.1126/science.1141752 17446353 10.1126/science.1141752

[4] Michaels SD, Himelblau E, Kim SY, Schomburg FM, Amasino RM. Integration of flowering signals in winter-annual *Arabidopsis*. Plant Physiol. 2005;137(1):149–56. doi: 10.1104/pp.104.052811 15618421 10.1104/pp.104.052811 PMC548846

[5] Yano M, Katayose Y, Ashikari M, Yamanouchi U, Monna L, Fuse T, et al *Hd1*, a major photoperiod sensitivity quantitative trait locus in rice, is closely related to the *Arabidopsis* flowering time gene *CONSTANS*. Plant Cell. 2000;12(12):2473–83. 11148291 10.1105/tpc.12.12.2473 PMC102231

[6] Kojima S, Takahashi Y, Kobayashi Y, Monna L, Sasaki T, Araki T, et al *Hd3a*, a rice ortholog of the *Arabidopsis FT* gene, promotes transition to flowering downstream of *Hd1* under short-day conditions. Plant Cell Physiol. 2002;43(10):1096–105. 12407188 10.1093/pcp/pcf156

[7] Griffiths S, Dunford RP, Coupland G, Laurie DA. The evolution of *CONSTANS*-like gene families in barley, rice, and *Arabidopsis*. Plant Physiol. 2003;131(4):1855–67. doi: 10.1104/pp.102.016188 12692345 10.1104/pp.102.016188 PMC166942

[8] Hayama R, Yokoi S, Tamaki S, Yano M, Shimamoto K. Adaptation of photoperiodic control pathways produces short-day flowering in rice. Nature. 2003;422(6933):719–22. doi: 10.1038/nature01549 12700762 10.1038/nature01549

[9] Nemoto Y, Kisaka M, Fuse T, Yano M, Ogihara Y. Characterization and functional analysis of three wheat genes with homology to the CONSTANS flowering time gene in transgenic rice. Plant J. 2003;36(1):82–93. 12974813 10.1046/j.1365-313x.2003.01859.x

[10] Faure S, Higgins J, Turner A, Laurie DA. The *FLOWERING LOCUS T*-like gene family in barley (*Hordeum vulgare*). Genetics. 2007;176(1):599–609. doi: 10.1534/genetics.106.069500 17339225 10.1534/genetics.106.069500 PMC1893030

[11] Danilevskaya ON, Meng X, Hou Z, Ananiev EV, Simmons CR. A genomic and expression compendium of the expanded *PEBP* gene family from maize. Plant Physiol. 2008;146(1):250–64. doi: 10.1104/pp.107.109538 17993543 10.1104/pp.107.109538 PMC2230559

[12] Chouard P. Vernalization and its relations to dormancy. Annu Rev Plant Physiol. 1960; 11: 191–238.

[13] Michaels SD, Amasino RM. *FLOWERING LOCUS C* encodes a novel MADS domain protein that acts as a repressor of flowering. Plant Cell. 1999;11(5):949–56. 10330478 10.1105/tpc.11.5.949 PMC144226

[14] Trevaskis B, Hemming MN, Dennis ES, Peacock WJ. The molecular basis of vernalization-induced flowering in cereals. Trends Plant Sci. 2007;12(8):352–7. doi: 10.1016/j.tplants.2007.06.010 17629542 10.1016/j.tplants.2007.06.010

[15] Zhao P, Liu P, Yuan G, Jia J, Li X, Qi D, et al New insights on drought stress response by global investigation of gene expression changes in Sheepgrass (*Leymus chinensis*). Front Plant Sci. 2016;7. 10.3389/fpls.2016.00954 PMC4928129 27446180

[16] Pankievicz V, Camilios-Neto D, Bonato P, Balsanelli E, Tadra-Sfeir M, Faoro H, et al RNA-seq transcriptional profiling of Herbaspirillum seropedicae colonizing wheat (*Triticum aestivum*) roots. Plant Mol Biol. 2016;90(6):589–603. doi: 10.1007/s11103-016-0430-6 26801330 10.1007/s11103-016-0430-6

[17] Zhang J, Liu W, Han H, Song L, Bai L, Gao Z, et al *De novo* transcriptome sequencing of *Agropyron cristatum* to identify available gene resources for the enhancement of wheat. Genomics. 2015;106(2):129–36. doi: 10.1016/j.ygeno.2015.04.003 25889708 10.1016/j.ygeno.2015.04.003

[18] Moore K, Moser LE, Vogel KP, Waller SS, Johnson B, Pedersen JF. Describing and quantifying growth stages of perennial forage grasses. Agro J. 1991;83(6):1073–7.

[19] Kopylova E, Noé L, Touzet H. SortMeRNA: fast and accurate filtering of ribosomal RNAs in metatranscriptomic data. Bioinformatics. 2012;28(24):3211–7. doi: 10.1093/bioinformatics/bts611 23071270 10.1093/bioinformatics/bts611

[20] Bolger AM, Lohse M, Usadel B. Trimmomatic: a flexible trimmer for Illumina sequence data. Bioinformatics. 2014:btu170. 10.1093/bioinformatics/btu170 PMC4103590 24695404

[21] Grabherr MG, Haas BJ, Yassour M, Levin JZ, Thompson DA, Amit I, et al Full-length transcriptome assembly from RNA-Seq data without a reference genome. Nat Biotechnol. 2011;29(7):644–52. doi: 10.1038/nbt.1883 21572440 10.1038/nbt.1883 PMC3571712

[22] Haas BJ, Papanicolaou A, Yassour M, Grabherr M, Blood PD, Bowden J, et al *De novo* transcript sequence reconstruction from RNA-seq using the Trinity platform for reference generation and analysis. Nat Protoc. 2013;8(8):1494–512. doi: 10.1038/nprot.2013.084 23845962 10.1038/nprot.2013.084 PMC3875132

[23] Davidson NM, Oshlack A. Corset: enabling differential gene expression analysis for *de novo* assembled transcriptomes. Genome Biol. 2014;15(7):1. 10.1186/s13059-014-0410-6 PMC4165373 25063469

[24] Langmead B, Salzberg SL. Fast gapped-read alignment with Bowtie 2. Nat Methods. 2012;9(4):357–9. doi: 10.1038/nmeth.1923 22388286 10.1038/nmeth.1923 PMC3322381

[25] Moriya Y, Itoh M, Okuda S, Yoshizawa AC, Kanehisa M. KAAS: an automatic genome annotation and pathway reconstruction server. Nucleic Acids Res. 2007;35(suppl 2):W182–W5. 17526522 10.1093/nar/gkm321 PMC1933193

[26] Aoki-Kinoshita KF, Kanehisa M. Gene annotation and pathway mapping in KEGG. Methods Mol Biol. 2007;396:71–91. doi: 10.1007/978-1-59745-515-2_6 18025687 10.1007/978-1-59745-515-2_6

[27] Tamura K, Stecher G, Peterson D, Filipski A, Kumar S. MEGA6: molecular evolutionary genetics analysis version 6.0. Mol Biol Evol. 2013;30(12):2725–9. doi: 10.1093/molbev/mst197 24132122 10.1093/molbev/mst197 PMC3840312

[28] Li B, Dewey CN. RSEM: accurate transcript quantification from RNA-Seq data with or without a reference genome. BMC Bioinformatics. 2011;12(1):1. 21816040 10.1186/1471-2105-12-323 PMC3163565

[29] Langmead B, Trapnell C, Pop M, Salzberg SL. Ultrafast and memory-efficient alignment of short DNA sequences to the human genome. Genome Biol. 2009;10(3):1. 10.1186/gb-2009-10-3-r25 PMC2690996 19261174

[30] Robinson MD, McCarthy DJ, Smyth GK. edgeR: a Bioconductor package for differential expression analysis of digital gene expression data. Bioinformatics. 2010;26(1):139–40. doi: 10.1093/bioinformatics/btp616 19910308 10.1093/bioinformatics/btp616 PMC2796818

[31] Benjamini Y, Yekutieli D. The control of the false discovery rate in multiple testing under dependency. Ann Stat. 2001;29(4):1165–88.

[32] Wu J, Mao X, Cai T, Luo J, Wei L. KOBAS server: a web-based platform for automated annotation and pathway identification. Nucleic Acids Res. 2006;34(suppl 2):W720–W4. 16845106 10.1093/nar/gkl167 PMC1538915

[33] Ledger S, Strayer C, Ashton F, Kay SA, Putterill J. Analysis of the function of two circadian‐regulated *CONSTANS‐LIKE* genes. Plant J. 2001;26(1):15–22. 11359606 10.1046/j.1365-313x.2001.01003.x

[34] Robson F, Costa MMR, Hepworth SR, Vizir I, Reeves PH, Putterill J, et al Functional importance of conserved domains in the flowering‐time gene *CONSTANS* demonstrated by analysis of mutant alleles and transgenic plants. Plant J. 2001;28(6):619–31. 11851908 10.1046/j.1365-313x.2001.01163.x

[35] Dornelas M, Rodriguez A. Identifying Eucalyptus expressed sequence tags related to *Arabidopsis* flowering-time pathway genes. Braz J Plant Physiol. 2005;17(2):255–66.

[36] Dornelas M, Rodriguez A. Evolutionary conservation of genes controlling flowering pathways between *Arabidopsis* and grasses In: Teixeira da Silva J, editor. Floriculture, ornamental and plant biotechnology. Global Science Books: London; 2006 pp. 272–279.

[37] Searle I, He Y, Turck F, Vincent C, Fornara F, Kröber S, et al The transcription factor FLC confers a flowering response to vernalization by repressing meristem competence and systemic signaling in *Arabidopsis*. Genes Dev. 2006;20(7):898–912. doi: 10.1101/gad.373506 16600915 10.1101/gad.373506 PMC1472290

[38] Dennis ES, Peacock WJ. Vernalization in cereals. J Bio. 2009;8(6):1. 10.1186/jbiol156 PMC2737375 19591652

[39] Distelfeld A, Li C, Dubcovsky J. Regulation of flowering in temperate cereals. Curr Opin Plant Biol. 2009;12(2):178–84. doi: 10.1016/j.pbi.2008.12.010 19195924 10.1016/j.pbi.2008.12.010

[40] Greenup A, Peacock WJ, Dennis ES, Trevaskis B. The molecular biology of seasonal flowering-responses in *Arabidopsis* and the cereals. Ann Bot. 2009;103(8):1165–72. doi: 10.1093/aob/mcp063 19304997 10.1093/aob/mcp063 PMC2685306

[41] Sasani S, Hemming MN, Oliver SN, Greenup A, Tavakkol-Afshari R, Mahfoozi S, et al The influence of vernalization and daylength on expression of flowering-time genes in the shoot apex and leaves of barley (*Hordeum vulgare*). J Exp Bot. 2009;60(7):2169–78. doi: 10.1093/jxb/erp098 19357429 10.1093/jxb/erp098 PMC2682508

[42] Preston JC, Kellogg EA. Reconstructing the evolutionary history of paralogous *APETALA1/FRUITFULL*-like genes in grasses (Poaceae). Genetics. 2006;174(1):421–37. doi: 10.1534/genetics.106.057125 16816429 10.1534/genetics.106.057125 PMC1569798

[43] Yan L, Loukoianov A, Blechl A, Tranquilli G, Ramakrishna W, SanMiguel P, et al The wheat *VRN2* gene is a flowering repressor down-regulated by vernalization. Science. 2004;303(5664):1640–4. doi: 10.1126/science.1094305 15016992 10.1126/science.1094305 PMC4737501

[44] Dubcovsky J, Chen C, Yan L. Molecular characterization of the allelic variation at the *VRN-H2* vernalization locus in barley. Mol Breed. 2005;15(4):395–407.

[45] Karsai I, Szűcs P, Mészáros K, Filichkina T, Hayes P, Skinner J, et al The *Vrn-H2* locus is a major determinant of flowering time in a facultative× winter growth habit barley (*Hordeum vulgare* L.) mapping population. Theor Appl Genet. 2005;110(8):1458–66. doi: 10.1007/s00122-005-1979-7 15834697 10.1007/s00122-005-1979-7

[46] Distelfeld A, Tranquilli G, Li C, Yan L, Dubcovsky J. Genetic and molecular characterization of the *VRN2* loci in tetraploid wheat. Plant Physiol. 2009;149(1):245–57. doi: 10.1104/pp.108.129353 19005084 10.1104/pp.108.129353 PMC2613703

[47] Yan L, Fu D, Li C, Blechl A, Tranquilli G, Bonafede M, et al The wheat and barley vernalization gene *VRN3* is an orthologue of *FT*. Proc Natl Acad Sci U S A. 2006;103(51):19581–6. doi: 10.1073/pnas.0607142103 17158798 10.1073/pnas.0607142103 PMC1748268

[48] Hemming MN, Peacock WJ, Dennis ES, Trevaskis B. Low-temperature and daylength cues are integrated to regulate *FLOWERING LOCUS T* in barley. Plant Physiol. 2008;147(1):355–66. doi: 10.1104/pp.108.116418 18359843 10.1104/pp.108.116418 PMC2330320

[49] Yan L, Helguera M, Kato K, Fukuyama S, Sherman J, Dubcovsky J. Allelic variation at the *VRN-1* promoter region in polyploid wheat. Theor Applied Genet. 2004;109(8):1677–86. 15480533 10.1007/s00122-004-1796-4

[50] Trevaskis B, Hemming MN, Peacock WJ, Dennis ES. *HvVRN2* responds to daylength, whereas HvVRN1 is regulated by vernalization and developmental status. Plant Physiol. 2006;140(4):1397–405. doi: 10.1104/pp.105.073486 16500994 10.1104/pp.105.073486 PMC1435809

[51] Sullivan JA, Deng XW. From seed to seed: the role of photoreceptors in *Arabidopsis* development. Dev Biol. 2003;260(2):289–97. 12921732 10.1016/s0012-1606(03)00212-4

[52] Sharrock RA, Quail PH. Novel phytochrome sequences in *Arabidopsis thaliana*: structure, evolution, and differential expression of a plant regulatory photoreceptor family. Genes Dev. 1989;3(11):1745–57. 2606345 10.1101/gad.3.11.1745

[53] Devlin PF, Patel SR, Whitelam GC. Phytochrome E influences internode elongation and flowering time in *Arabidopsis*. Plant Cell. 1998;10(9):1479–87. 9724694 10.1105/tpc.10.9.1479 PMC144080

[54] Briggs WR, Huala E. Blue-light photoreceptors in higher plants. Annu Rev Cell Dev Biol. 1999;15(1):33–62. 10611956 10.1146/annurev.cellbio.15.1.33

[55] Devlin PF, Robson PR, Patel SR, Goosey L, Sharrock RA, Whitelam GC. Phytochrome D acts in the shade-avoidance syndrome in *Arabidopsis* by controlling elongation growth and flowering time. Plant Physiol. 1999;119(3):909–16. 10069829 10.1104/pp.119.3.909 PMC32105

[56] Dunlap JC. Molecular bases for circadian clocks. Cell. 1999;96(2):271–90. 9988221 10.1016/s0092-8674(00)80566-8

[57] Zagotta MT, Hicks KA, Jacobs CI, Young JC, Hangarter RP, Meeks‐Wagner DR. The *Arabidopsis ELF3* gene regulates vegetative photomorphogenesis and the photoperiodic induction of flowering. Plant J. 1996;10(4):691–702. 8893545 10.1046/j.1365-313x.1996.10040691.x

[58] Wang Z-Y, Kenigsbuch D, Sun L, Harel E, Ong MS, Tobin EM. A Myb-related transcription factor is involved in the phytochrome regulation of an *Arabidopsis Lhcb* gene. Plant Cell. 1997;9(4):491–507. doi: 10.1105/tpc.9.4.491 9144958 10.1105/tpc.9.4.491 PMC156934

[59] Schaffer R, Ramsay N, Samach A, Corden S, Putterill J, Carré IA, et al The late elongated hypocotyl mutation of *Arabidopsis* disrupts circadian rhythms and the photoperiodic control of flowering. Cell. 1998;93(7):1219–29. 9657154 10.1016/s0092-8674(00)81465-8

[60] Wang Z-Y, Tobin EM. Constitutive expression of the *CIRCADIAN CLOCK ASSOCIATED 1* (*CCA1*) gene disrupts circadian rhythms and suppresses its own expression. Cell. 1998;93(7):1207–17. 9657153 10.1016/s0092-8674(00)81464-6

[61] Somers DE, Webb A, Pearson M, Kay SA. The short-period mutant, *toc1-1*, alters circadian clock regulation of multiple outputs throughout development in *Arabidopsis thaliana*. Development. 1998;125(3):485–94. 9425143 10.1242/dev.125.3.485

[62] Fowler S, Lee K, Onouchi H, Samach A, Richardson K, Morris B, et al *GIGANTEA*: a circadian clock‐controlled gene that regulates photoperiodic flowering in *Arabidopsis* and encodes a protein with several possible membrane‐spanning domains. EMBO J. 1999;18(17):4679–88. doi: 10.1093/emboj/18.17.4679 10469647 10.1093/emboj/18.17.4679 PMC1171541

[63] Park DH, Somers DE, Kim YS, Choy YH, Lim HK, Soh MS, et al Control of circadian rhythms and photoperiodic flowering by the *Arabidopsis GIGANTEA* gene. Science. 1999;285(5433):1579–82. 10477524 10.1126/science.285.5433.1579

[64] Suárez-López P, Wheatley K, Robson F, Onouchi H, Valverde F, Coupland G. *CONSTANS* mediates between the circadian clock and the control of flowering in *Arabidopsis*. Nature. 2001;410(6832):1116–20. doi: 10.1038/35074138 11323677 10.1038/35074138

[65] Yanovsky MJ, Kay SA. Molecular basis of seasonal time measurement in *Arabidopsis*. Nature. 2002;419(6904):308–12. doi: 10.1038/nature00996 12239570 10.1038/nature00996

[66] Abe M, Kobayashi Y, Yamamoto S, Daimon Y, Yamaguchi A, Ikeda Y, et al FD, a bZIP protein mediating signals from the floral pathway integrator FT at the shoot apex. Science. 2005;309(5737):1052–6. doi: 10.1126/science.1115983 16099979 10.1126/science.1115983

[67] Wigge PA, Kim MC, Jaeger KE, Busch W, Schmid M, Lohmann JU, et al Integration of spatial and temporal information during floral induction in *Arabidopsis*. Science. 2005;309(5737):1056–9. doi: 10.1126/science.1114358 16099980 10.1126/science.1114358

[68] Valverde F, Mouradov A, Soppe W, Ravenscroft D, Samach A, Coupland G. Photoreceptor regulation of CONSTANS protein in photoperiodic flowering. Science. 2004;303(5660):1003–6. doi: 10.1126/science.1091761 14963328 10.1126/science.1091761

[69] Griffiths S, Dunford RP, Coupland G, Laurie DA. The evolution of CONSTANS-like gene families in barley, rice, and *Arabidopsis*. Plant Physiol. 2003;131(4):1855–67. doi: 10.1104/pp.102.016188 12692345 10.1104/pp.102.016188 PMC166942

[70] Turck F, Fornara F, Coupland G. Regulation and identity of florigen: *FLOWERING LOCUS T* moves center stage. Annu Rev Plant Biol. 2008;59:573–94. doi: 10.1146/annurev.arplant.59.032607.092755 18444908 10.1146/annurev.arplant.59.032607.092755

[71] Tamaki S, Matsuo S, Wong HL, Yokoi S, Shimamoto K. Hd3a protein is a mobile flowering signal in rice. Science. 2007;316(5827):1033–6. doi: 10.1126/science.1141753 17446351 10.1126/science.1141753

