# Supplementary material for: RNA-Seq analysis of gene expression for floral development in crested wheatgrass (Agropyron cristatum L.)
Source: PLoS One. 2017 May 22;12(5):e0177417. doi: 10.1371/journal.pone.0177417 (PMC5439701; doi:10.1371/journal.pone.0177417)
Supplement: S1 File — (DOCX) [file pone.0177417.s001.docx]

| Year | Plant introduction |  | Head count(number of heads/date) | Flowering time | Maturity |
| --- | --- | --- | --- | --- | --- |
| 2015 | PI 598641 |  | 20 | 03-Jun-2015 | Early |
| 2015 | W625134 |  | 20 | 03-Jun-2015 | Early |
| 2015 | PI 439914 |  | 20 | 03-Jun-2015 | Early |
| 2015 | PI 439914 |  | 20 | 03-Jun-2015 | Early |
| 2016 | PI 598641 |  | >20 | 26-May-2016 | Early |
| 2016 | W625134 |  | >20 | 26-May-2016 | Early |
| 2016 | PI 439914 |  | >20 | 26-May-2016 | Early |
| 2016 | PI 439914 |  | >20 | 26-May-2016 | Early |

**S1 File** **The flowering date of the four selected tetraploid accessions of crested wheatgrass in year 2015 and 2016 at Saskatoon, SK, Canada.**
