# Supplementary material for: RNA-Seq analysis of gene expression for floral development in crested wheatgrass (Agropyron cristatum L.)
Source: PLoS One. 2017 May 22;12(5):e0177417. doi: 10.1371/journal.pone.0177417 (PMC5439701; doi:10.1371/journal.pone.0177417)
Supplement: S2 File — (DOCX) [file pone.0177417.s002.docx]

**S2 File. Primer pairs used for qRT-PCR analysis**

| No. |  | Primer Name | Sequence (5'-3') |
| --- | --- | --- | --- |
| 1 |  | DN56531F2 | AGGATGTACAATTGACCTATCACC |
|  |  | DN56531R2 | ATTCGTGGCTGGCTTCTTAT |
| 2 |  | DN60312F1 | CTGCTCCTGGTGATGAATGT |
|  |  | DN60312R1 | AGCACCTCGTAGAGACAGAA |
| 3 |  | DN53956F1 | CTGCAAGGTTGATGTCTGATCTA |
|  |  | DN53956R1 | ACAGACATAAGTACCGGCAAAG |
| 4 |  | DN59278F2 | GCATAAGTTGTGGGTGCTTAC |
|  |  | DN59278R2 | TAGATGGTGTGGTGGAGTTATG |
| 5 |  | DN55669F2 | TTGGTGCCTGTCTCAGGTATAG |
|  |  | DN55669R2 | CTCAAGTGTCTTCTCCGCATTG |
| 6 |  | DN53048F2 | GAACACGAAGCGGTGGAT |
|  |  | DN53048R2 | GCACTGGCTTGTGACAGATA |
| 7 |  | DN60312F2 | TTGGATCCAGTACAGCTCTTTC |
|  |  | DN60312R2 | CTCATCGTCTTCTCCACCAAG |
| 8 |  | DN59116F1 | TGTGACCAGCAGCTAAAGTC |
|  |  | DN59116R1 | CCAGAGAACGAGAGGGAAATAC |
| 9 |  | DN61161F2 | GGTAAGGCCGAAGGTTGTTA |
|  |  | DN61161R2 | CCAGTAGGTAAGCCCACAAA |
| 10 |  | DN56706F1 | CCGCAATTTCCCACCATTAAG |
|  |  | DN56706R1 | AGCTACCAGCCACAGAATTATC |
| 11 |  | DN60175F1 | GAAGACGGCATACGAGATGAT |
|  |  | DN60175R1 | CTAGCCTTGTCCTGAGGTTATT |
| 12 |  | DN61626F2 | CGAGCAGCCATACTCATTGT |
|  |  | DN61626R2 | GACTAGATTGACCCGCATACTG |
| 13 |  | DN55370F2 | GGTTCACCAGCAGCATCAA |
|  |  | DN55370R2 | CTCTGGGAGCACAACAACAA |
| 14 |  | DN55848F2 | GGAGAGCAAGGGTGTTGTTAT |
|  |  | DN55848R2 | CCTGTAACTATCGGTCGTGTTC |
| 15 |  | DN56923F2 | CCACCGAGATCTACACTCTTTC |
|  |  | DN56923R2 | CTATCCTGTGCCGTGGATTT |
| 16 |  | DN36907F2 | ACACACTCTTCTCCCTCATTTG |
|  |  | DN36907R2 | CATTAGTCCTGTGGATGGTGTAG |
