# Supplementary material for: RNA-Seq analysis of gene expression for floral development in crested wheatgrass (Agropyron cristatum L.)
Source: PLoS One. 2017 May 22;12(5):e0177417. doi: 10.1371/journal.pone.0177417 (PMC5439701; doi:10.1371/journal.pone.0177417)
Supplement: S8 File — (DOCX) [file pone.0177417.s008.docx]

**S8 File. A summary of 298 KEGG pathways identified in the crested wheatgrass flowering initiation and development transcriptome.**

ko01100 Metabolic pathways (830)

ko01110 Biosynthesis of secondary metabolites (384)

ko01130 Biosynthesis of antibiotics (188)

ko01120 Microbial metabolism in diverse environments (133)

ko03010 Ribosome (132)

ko03040 Spliceosome (100)

ko01230 Biosynthesis of amino acids (99)

ko03013 RNA transport (93)

ko00230 Purine metabolism (89)

ko01200 Carbon metabolism (87)

ko00190 Oxidative phosphorylation (86)

ko04141 Protein processing in endoplasmic reticulum (76)

ko00240 Pyrimidine metabolism (74)

ko04120 Ubiquitin mediated proteolysis (58)

ko04110 Cell cycle (58)

ko03008 Ribosome biogenesis in eukaryotes (57)

ko04144 Endocytosis (56)

ko04111 Cell cycle - yeast (53)

ko03018 RNA degradation (49)

ko03015 mRNA surveillance pathway (48)

ko00195 Photosynthesis (41)

ko04113 Meiosis - yeast (40)

ko04075 Plant hormone signal transduction (39)

ko00520 Amino sugar and nucleotide sugar metabolism (39)

ko05203 Viral carcinogenesis (38)

ko03420 Nucleotide excision repair (37)

ko04146 Peroxisome (36)

ko04142 Lysosome (36)

ko00270 Cysteine and methionine metabolism (35)

ko00564 Glycerophospholipid metabolism (35)

ko00260 Glycine, serine and threonine metabolism (34)

ko04016 MAPK signaling pathway - plant (33)

ko03050 Proteasome (33)

ko03440 Homologous recombination (33)

ko03460 Fanconi anemia pathway (32)

ko03030 DNA replication (32)

ko00010 Glycolysis / Gluconeogenesis (32)

ko00860 Porphyrin and chlorophyll metabolism (31)

ko04114 Oocyte meiosis (31)

ko00510 N-Glycan biosynthesis (31)

ko00900 Terpenoid backbone biosynthesis (30)

ko03022 Basal transcription factors (30)

ko03020 RNA polymerase (30)

ko00500 Starch and sucrose metabolism (30)

ko04145 Phagosome (29)

ko01210 2-Oxocarboxylic acid metabolism (28)

ko00250 Alanine, aspartate and glutamate metabolism (27)

ko04150 mTOR signaling pathway (27)

ko03060 Protein export (26)

ko00620 Pyruvate metabolism (26)

ko00970 Aminoacyl-tRNA biosynthesis (26)

ko00630 Glyoxylate and dicarboxylate metabolism (26)

ko00561 Glycerolipid metabolism (25)

ko00710 Carbon fixation in photosynthetic organisms (25)

ko03410 Base excision repair (25)

ko04626 Plant-pathogen interaction (25)

ko00330 Arginine and proline metabolism (24)

ko04152 AMPK signaling pathway (24)

ko00513 Various types of N-glycan biosynthesis (24)

ko04151 PI3K-Akt signaling pathway (23)

ko00562 Inositol phosphate metabolism (23)

ko04721 Synaptic vesicle cycle (23)

ko00400 Phenylalanine, tyrosine and tryptophan biosynthesis (23)

ko04914 Progesterone-mediated oocyte maturation (22)

ko00280 Valine, leucine and isoleucine degradation (21)

ko03430 Mismatch repair (21)

ko00220 Arginine biosynthesis (21)

ko00563 Glycosylphosphatidylinositol (GPI)-anchor biosynthesis (21)

ko01212 Fatty acid metabolism (21)

ko04712 Circadian rhythm - plant (20)

ko00051 Fructose and mannose metabolism (20)

ko00020 Citrate cycle (TCA cycle) (20)

ko04070 Phosphatidylinositol signaling system (20)

ko04130 SNARE interactions in vesicular transport (19)

ko00130 Ubiquinone and other terpenoid-quinone biosynthesis (19)

ko04068 FoxO signaling pathway (18)

ko00100 Steroid biosynthesis (18)

ko00680 Methane metabolism (18)

ko00350 Tyrosine metabolism (18)

ko04910 Insulin signaling pathway (18)

ko00030 Pentose phosphate pathway (17)

ko04071 Sphingolipid signaling pathway (17)

ko00940 Phenylpropanoid biosynthesis (17)

ko00480 Glutathione metabolism (17)

ko00410 beta-Alanine metabolism (16)

ko04623 Cytosolic DNA-sensing pathway (16)

ko00052 Galactose metabolism (16)

ko04310 Wnt signaling pathway (16)

ko00906 Carotenoid biosynthesis (16)

ko04810 Regulation of actin cytoskeleton (16)

ko00770 Pantothenate and CoA biosynthesis (16)

ko04066 HIF-1 signaling pathway (16)

ko00360 Phenylalanine metabolism (16)

ko00053 Ascorbate and aldarate metabolism (15)

ko04922 Glucagon signaling pathway (15)

ko04919 Thyroid hormone signaling pathway (15)

ko05323 Rheumatoid arthritis (15)

ko04140 Autophagy (15)

ko04139 Mitophagy - yeast (14)

ko04211 Longevity regulating pathway (14)

ko00941 Flavonoid biosynthesis (14)

ko00790 Folate biosynthesis (14)

ko00640 Propanoate metabolism (14)

ko00920 Sulfur metabolism (14)

ko00600 Sphingolipid metabolism (14)

ko04621 NOD-like receptor signaling pathway (14)

ko04666 Fc gamma R-mediated phagocytosis (13)

ko05134 Legionellosis (13)

ko02024 Quorum sensing (13)

ko04011 MAPK signaling pathway - yeast (13)

ko00592 alpha-Linolenic acid metabolism (13)

ko04931 Insulin resistance (13)

ko00040 Pentose and glucuronate interconversions (13)

ko00910 Nitrogen metabolism (13)

ko00460 Cyanoamino acid metabolism (13)

ko04213 Longevity regulating pathway - multiple species (12)

ko04115 p53 signaling pathway (12)

ko04013 MAPK signaling pathway - fly (12)

ko04722 Neurotrophin signaling pathway (12)

ko00310 Lysine degradation (11)

ko00071 Fatty acid degradation (11)

ko00196 Photosynthesis - antenna proteins (11)

ko04966 Collecting duct acid secretion (11)

ko00730 Thiamine metabolism (11)

ko04612 Antigen processing and presentation (11)

ko00340 Histidine metabolism (11)

ko00760 Nicotinate and nicotinamide metabolism (11)

ko00061 Fatty acid biosynthesis (11)

ko00650 Butanoate metabolism (10)

ko00380 Tryptophan metabolism (10)

ko00720 Carbon fixation pathways in prokaryotes (10)

ko00290 Valine, leucine and isoleucine biosynthesis (10)

ko04010 MAPK signaling pathway (10)

ko04014 Ras signaling pathway (10)

ko04728 Dopaminergic synapse (10)

ko04921 Oxytocin signaling pathway (10)

ko04350 TGF-beta signaling pathway (10)

ko00670 One carbon pool by folate (10)

ko01040 Biosynthesis of unsaturated fatty acids (9)

ko00950 Isoquinoline alkaloid biosynthesis (9)

ko04122 Sulfur relay system (9)

ko04022 cGMP-PKG signaling pathway (9)

ko00511 Other glycan degradation (9)

ko00300 Lysine biosynthesis (9)

ko02020 Two-component system (9)

ko00450 Selenocompound metabolism (9)

ko04072 Phospholipase D signaling pathway (9)

ko00750 Vitamin B6 metabolism (8)

ko00565 Ether lipid metabolism (8)

ko00780 Biotin metabolism (8)

ko05211 Renal cell carcinoma (8)

ko00960 Tropane, piperidine and pyridine alkaloid biosynthesis (8)

ko04024 cAMP signaling pathway (8)

ko04341 Hedgehog signaling pathway - fly (8)

ko01521 EGFR tyrosine kinase inhibitor resistance (8)

ko05130 Pathogenic Escherichia coli infection (8)

ko04727 GABAergic synapse (8)

ko04390 Hippo signaling pathway (8)

ko00540 Lipopolysaccharide biosynthesis (8)

ko04020 Calcium signaling pathway (8)

ko03320 PPAR signaling pathway (8)

ko03450 Non-homologous end-joining (8)

ko05322 Systemic lupus erythematosus (8)

ko04261 Adrenergic signaling in cardiomyocytes (8)

ko04391 Hippo signaling pathway - fly (7)

ko00062 Fatty acid elongation (7)

ko00905 Brassinosteroid biosynthesis (7)

ko04510 Focal adhesion (7)

ko04915 Estrogen signaling pathway (7)

ko00740 Riboflavin metabolism (7)

ko00904 Diterpenoid biosynthesis (7)

ko00073 Cutin, suberine and wax biosynthesis (7)

ko04064 NF-kappa B signaling pathway (7)

ko04710 Circadian rhythm (7)

ko04330 Notch signaling pathway (7)

ko05145 Toxoplasmosis (7)

ko01522 Endocrine resistance (7)

ko00261 Monobactam biosynthesis (6)

ko05014 Amyotrophic lateral sclerosis (ALS) (6)

ko04620 Toll-like receptor signaling pathway (6)

ko04978 Mineral absorption (6)

ko04724 Glutamatergic synapse (6)

ko04920 Adipocytokine signaling pathway (6)

ko04622 RIG-I-like receptor signaling pathway (6)

ko00514 Other types of O-glycan biosynthesis (6)

ko00945 Stilbenoid, diarylheptanoid and gingerol biosynthesis (6)

ko05214 Glioma (6)

ko00590 Arachidonic acid metabolism (6)

ko04720 Long-term potentiation (6)

ko03070 Bacterial secretion system (6)

ko04961 Endocrine and other factor-regulated calcium reabsorption (6)

ko04112 Cell cycle - Caulobacter (5)

ko04624 Toll and Imd signaling pathway (5)

ko04062 Chemokine signaling pathway (5)

ko04015 Rap1 signaling pathway (5)

ko04917 Prolactin signaling pathway (5)

ko00660 C5-Branched dibasic acid metabolism (5)

ko00908 Zeatin biosynthesis (5)

ko00627 Aminobenzoate degradation (5)

ko04662 B cell receptor signaling pathway (5)

ko04962 Vasopressin-regulated water reabsorption (5)

ko04360 Axon guidance (5)

ko04916 Melanogenesis (5)

ko04380 Osteoclast differentiation (5)

ko04370 VEGF signaling pathway (5)

ko00909 Sesquiterpenoid and triterpenoid biosynthesis (5)

ko04540 Gap junction (5)

ko04918 Thyroid hormone synthesis (5)

ko01523 Antifolate resistance (5)

ko05020 Prion diseases (5)

ko04340 Hedgehog signaling pathway (5)

ko00531 Glycosaminoglycan degradation (5)

ko05133 Pertussis (5)

ko00643 Styrene degradation (5)

ko04012 ErbB signaling pathway (5)

ko04912 GnRH signaling pathway (5)

ko05146 Amoebiasis (4)

ko05140 Leishmaniasis (4)

ko00521 Streptomycin biosynthesis (4)

ko00980 Metabolism of xenobiotics by cytochrome P450 (4)

ko00830 Retinol metabolism (4)

ko02010 ABC transporters (4)

ko04550 Signaling pathways regulating pluripotency of stem cells (4)

ko00982 Drug metabolism - cytochrome P450 (4)

ko04975 Fat digestion and absorption (4)

ko04659 Th17 cell differentiation (4)

ko05218 Melanoma (4)

ko04723 Retrograde endocannabinoid signaling (4)

ko00140 Steroid hormone biosynthesis (4)

ko04650 Natural killer cell mediated cytotoxicity (4)

ko00591 Linoleic acid metabolism (4)

ko04664 Fc epsilon RI signaling pathway (4)

ko04713 Circadian entrainment (3)

ko00603 Glycosphingolipid biosynthesis - globo and isoglobo series (3)

ko05204 Chemical carcinogenesis (3)

ko01220 Degradation of aromatic compounds (3)

ko04725 Cholinergic synapse (3)

ko04974 Protein digestion and absorption (3)

ko04933 AGE-RAGE signaling pathway in diabetic complications (3)

ko04726 Serotonergic synapse (3)

ko04614 Renin-angiotensin system (3)

ko00625 Chloroalkane and chloroalkene degradation (3)

ko04630 Jak-STAT signaling pathway (3)

ko00072 Synthesis and degradation of ketone bodies (3)

ko04320 Dorso-ventral axis formation (3)

ko00440 Phosphonate and phosphinate metabolism (3)

ko00430 Taurine and hypotaurine metabolism (3)

ko04973 Carbohydrate digestion and absorption (3)

ko04668 TNF signaling pathway (3)

ko04750 Inflammatory mediator regulation of TRP channels (2)

ko04744 Phototransduction (2)

ko00785 Lipoic acid metabolism (2)

ko00903 Limonene and pinene degradation (2)

ko04658 Th1 and Th2 cell differentiation (2)

ko04976 Bile secretion (2)

ko04964 Proximal tubule bicarbonate reclamation (2)

ko00232 Caffeine metabolism (2)

ko00401 Novobiocin biosynthesis (2)

ko00902 Monoterpenoid biosynthesis (2)

ko00550 Peptidoglycan biosynthesis (2)

ko05217 Basal cell carcinoma (2)

ko05410 Hypertrophic cardiomyopathy (HCM) (2)

ko00362 Benzoate degradation (2)

ko04611 Platelet activation (2)

ko04960 Aldosterone-regulated sodium reabsorption (2)

ko04711 Circadian rhythm - fly (2)

ko04740 Olfactory transduction (2)

ko00626 Naphthalene degradation (2)

ko00901 Indole alkaloid biosynthesis (2)

ko02025 Biofilm formation - Pseudomonas aeruginosa (2)

ko00944 Flavone and flavonol biosynthesis (2)

ko01502 Vancomycin resistance (2)

ko00402 Benzoxazinoid biosynthesis (2)

ko00604 Glycosphingolipid biosynthesis - ganglio series (2)

ko04923 Regulation of lipolysis in adipocytes (1)

ko04745 Phototransduction - fly (1)

ko00601 Glycosphingolipid biosynthesis - lacto and neolacto series (1)

ko04925 Aldosterone synthesis and secretion (1)

ko00281 Geraniol degradation (1)

ko00471 D-Glutamine and D-glutamate metabolism (1)

ko00523 Polyketide sugar unit biosynthesis (1)

ko00624 Polycyclic aromatic hydrocarbon degradation (1)

ko01503 Cationic antimicrobial peptide (CAMP) resistance (1)

ko01053 Biosynthesis of siderophore group nonribosomal peptides (1)

ko04971 Gastric acid secretion (1)

ko00966 Glucosinolate biosynthesis (1)

ko00623 Toluene degradation (1)

ko01051 Biosynthesis of ansamycins (1)

ko05111 Biofilm formation - Vibrio cholerae (1)

ko04670 Leukocyte transendothelial migration (1)

ko00364 Fluorobenzoate degradation (1)

ko00524 Neomycin, kanamycin and gentamicin biosynthesis (1)

ko00254 Aflatoxin biosynthesis (1)

ko00363 Bisphenol degradation (1)

ko00965 Betalain biosynthesis (1)

ko00361 Chlorocyclohexane and chlorobenzene degradation (1)

ko00791 Atrazine degradation (1)
